# A case–control study to predict the risk of gestational diabetes mellitus by initial fasting blood sugar or past gestational history

**DOI:** 10.18502/ijrm.v19i4.9064

**Published:** 2021-04-22

**Authors:** Tahmineh Ezazi Bojnordi, Sedigheh Hantoushzadeh, Masomeh Sabzevary, Zahra Heidari

**Affiliations:** ^1^Department of Obstetrics and Gynecology, Loghman Hospital, Shahid Beheshti University of Medical Sciences, Tehran, Iran.; ^2^Department of Gynecology and Obstetrics, School of Medicine, Tehran University of Medical Sciences, Tehran, Iran.; ^3^Department of Histology, Genetic of Non-communicable Diseases Research Center, Faculty of Medicine, Zahedan University of Medical Sciences, Zahedan, Iran.

**Keywords:** Diabetes Mellitus, Gestational, Blood glucose, Risk factor.

## Abstract

**Background:**

Gestational diabetes mellitus (GDM) deserves proper prevention, diagnosis, and management due to healthcare implications from both maternal and fetal concerns.

**Objective:**

To evaluate the rate and investigate the risk factors for developing GDM.

**Materials and Methods:**

In this case-control, universal screening for GDM between 24 and 28 wk of gestation was performed in 613 pregnant women attending a prenatal clinic in Tehran who were followed-up until delivery between March 2017 to March 2018. Of the 613 women, 143 had GDM and 470 had normal glucose tolerance test as the primary diagnosis. Some GDM risk factors were compared in two groups.

**Results:**

Impaired glucose tolerance test was detected in 143 (23.3%) patients. Prevalence of GDM was higher in the first-trimester fasting blood sugar (FBS) > 90 qmg/dl group (p < 0.001). Comparison of the GDM and the normal glucose tolerance test groups demonstrated significant differences in maternal age, first-trimester FBS, third-trimester vitamin D level, maternal platelet count, maternal body mass index (BMI) (before 12 wk of gestation), weight gain during pregnancy, and the history of gestational complications in previous pregnancy (p < 0.01). In logistic regression, GDM was independently associated with older maternal age, higher first-trimester FBS, the history of gestational complications in previous pregnancy, lower third-trimester vitamin D level, and higher maternal platelet count (p < 0.01).

**Conclusion:**

Both patients with higher initial FBS and the history of gestational complications in previous pregnancy should be considered high risk for GDM and screened earlier.

## 1. Introduction

Gestational diabetes mellitus (GDM) is defined as the glucose intolerance that is started or diagnosed essentially during the pregnancy (1, 2). The prevalence of GDM is variable depending on the area of study, population, methods of data collection, selection and diagnosis criteria, and screening programs. This condition occurs in 1-14% of all pregnancies in the United States. In Iran, the prevalence of the disease varies from 1.3 to 18.8% (3-5).

It leads to large for gestational age and metabolic disorders in infants, and preeclampsia and hypertension in mothers. Due to short- and long-term healthcare implications from both maternal and fetal concerns, GDM deserves proper prevention, diagnosis, and management. Factors that influence the rising prevalence of GDM include increased maternal age at the time of conception, sedentary life, obesity, and diabetes epidemics (4, 6). It is well-known that early diagnosis and management of women with risk of GDM, might diminish maternal and neonatal morbidity (7, 8).

Although enquiring about the risk factors associated with GDM is the cornerstone of any clinical assessment, the specificity and sensitivity of history for the diagnosis of GDM are very low and insufficient, it would be appropriate to recommend adding other factors for universal screening of early GDM diagnosis (5).

A previous obstetric history is reported as traditional and most often as a risk factor for GDM (9). In addition, a previous history of pregnancy complications including preeclampsia, hypertension, intra uterus growth retardation (IUGR), and GDM have common risk factors consisting of increased maternal age, nulliparity, multiple gestation pregnancies, and an increased pre-pregnancy body mass index (BMI). vascular endothelial dysfunction is considered to be the underlying pathophysiology of these disorders (10).

Fasting plasma glucose (FPG) is convenient to administer, nicely tolerated, affordable, reliable, reproducible, and has been reported to fluctuate very little during gestation. So, there are certain advantages of its use as a screening test for GDM than the glucose challenge test (11). Notwithstanding the excessive incidence of GDM in the far East population, few studies have targeted at the predictive risk factors for GDM, and records about factors related to GDM incidence is confined. Additional studies are needed to validate previous research on the factors associated with GDM.

Consequently, this survey was designed to investigate the risk factors associated with developing GDM and specifically to assess the significance of previous history of pregnancy complications and fasting blood sugar (FBS) level during first trimester to predict GDM.

## 2. Materials and Methods

This case-control study is a retrospective file review of women who received prenatal care at a prenatal clinic in Imam Khomeini Hospital, Tehran, Iran. Accordingly, our survey population was a high-risk group (because we had more pregnant women with gestational diabetic, growth retardation, hypertension and etc. than the normal papulation). Patients' medical records were utilized to create reports of all women who received prenatal care between March 2017 and March 2018. The inclusion criteria were: 1) maternal age ≥ 17 yr; 2) confirmed singleton pregnancy of < 16 wk gestation; and 3) planned to receive ongoing prenatal care in our prenatal health center. On the other hand, the exclusion criteria included: 1) twin or multifetal pregnancy; 2) maternal immune-deficiency diseases; 3) women's medical records indicating impaired glucose tolerance test (GTT), and/or pre-GDM; and 4) lack of data on the GTT, first-trimester FPG level, antenatal visits, or child delivery. In this survey, if the screening at the first prenatal visit showed An FBS ≥ 126 mg/dl, the case was also excluded.

At the first prenatal visit risk assessment was performed for all pregnant women. High-risk women for GDM (positive family history disease [FHD], age > 35 yr, prepregnancy obesity, personal previous history of GDM, previous macrosomia or glycosuria) were screened as soon as possible. High-risk women were retested at 24-28 wk if negative at the first visit. Women who were not at high risk for GDM at 24-28 wks' gestation had screening test by the three steps 75gr oral GTT (75 gr 2hOGTT) (3).

Patients underwent a 75 gr 2hOGTT after an appropriate three-day carbohydrate load and overnight fasting of at least 8 hr. Blood sugar (BS) was measured in fasting, and at 1 and 2 hr after the 75 gr oral glucose intake. GDM was diagnosed based on International association of diabetes and pregnancy study groups guidelines: any abnormal value equal or greater than knowing threshold values was considered GDM (FBS ≥ 92 mg/dl [5.1 mmol/L]; 1 hr BS ≥ 180 mg/dl [10.0 mmol/L)] 2 hr BS ≥ 153 mg/dl [8.5 mmol/L]) (one-step strategy according to American Diabetic Association) (12).

Moreover, serum glucose concentration was measured by the glucose oxidase method in an auto-analyzer (Roche Diagnostics). In the GDM group, nutrition counseling for the initiation of diabetic diet was conducted and a 2-wk observation was planned. Then, FBS and (2 hr post prandial BS) 2hppBS were checked. If FBS ≤ 92 mg/dl and 2hppBS ≤ 120 mg/dl, they were classified as class A1 GDM and controlled by diet and were followed by every 2 wk FBS and 2hppBS checking. If FBS > 92 mg/dl and/or 2hppBS > 120 mg/dl, they were classified as class A2 GDM and drug therapy (insulin or metformin) was considered (13).

In total, 701 medical records were collected. After review, 46 were excluded due to lack of data on the GTT, antenatal visits, or child delivery. Furthermore, 16 records with the diagnosis of type 1 or 2 diabetes and 26 records with pre-gestational diabetes were excluded. Eventually, 613 patients were included in the study of which 143 had GDM and 470 had normal glucose tolerance test (NGT) as the primary diagnosis. BMI (weight [kg] /height [m${}^{2}$]) was measured at 12 wks' gestation or earlier in this pregnancy. Every included participant had been asked about their use of vitamin and mineral supplements at each prenatal visit, and any multivitamin supplementation was mentioned. Data on the type of hyperglycemia control (nutrition, insulin, or metformin) were collected. Women who did not have GDM until the third trimester were followed-up monthly, after which till delivery fortnightly. For the GDM group, during the first and second trimesters antenatal care involved fortnightly visits, then weekly in the third trimester, if identified early. The GDM group was advised to obey diabetic diet based on nutrition counseling and exercise for at least 30 min three times weekly. They were under tight controls of BS during their pregnancies (FBS ≤ 92 mg/dl and 2hppBS ≤ 120 mg/dl).

Trained midwives had collected data in each prenatal visit and for all pregnant women included in the study obtained anthropometric and demographic data by an established questionnaire form. At each visit, mother's weight gain was recorded. All eligible pregnant women replied to a structured questionnaire about age, their past obstetric history (e.g., preeclampsia, hypertension, IUGR, preterm labor [PTL], Preterm premature rupture of the membranes [PPROM], placenta abruption and GDM), family history of diabetes in first degree relatives, parity and number of pregnancies. Physical examinations of the pregnant women were performed and arterial blood pressure and weight were recorded. First- and third-trimester laboratory data (FBS, thyroid stimulating hormone [TSH], hemoglobin, platelet, and Vitamin D) were obtained. The gestational age of 12 wk was calculated through sonography.

Moreover, pregnant women were followed-up until delivery for poor obstetric and neonatal outcomes. Maternal weight gain in each trimester during pregnancy and obstetric outcomes (gender of newborn, birth weight, type of delivery [caesarean section or normal vaginal delivery], PTL, IUGR, preeclampsia, and PPROM) were recorded.

The prevalence of GDM were compared between the groups of first trimester FBS ≤ 90 mg/dl and FBS > 90. GDM and NGT mothers were compared for the prevalence of different factors including, maternal age, first-trimester FBS, first and third-trimester vitamin D levels, maternal platelet count, maternal BMI (before the 12^th^ wk of gestation), weight gain during the pregnancy, nuchal translucency (NT), anemia, TSH, family history of diabetes mellitus, infant sex and infant weigh, prevalence of pregnancy complications (namely, PTL, PROM, preeclampsia, and IUGR), and the history of gestational complications in previous pregnancies (e.g., preeclampsia, hypertension, IUGR, PTL, placenta abruption, and GDM).

### Ethical considerations

The study was approved by the ethics committee of the Imam Khomeini Hospital, Tehran University of Medical Sciences, Tehran, Iran (Code: IR.TUMS.IKHC.RCE.1396.3435). A written informed consent was obtained from all patients about their anonymous and voluntary participation. They had been additionally ensured that the results of the study would be confidential and advantageous to them or other pregnant women.

### Statistical analysis

Data were analyzed using the version 18.0 of SPSS for Windows (SPSS Inc., Chicago, Illinois, USA). In determining the prevalence and mean values descriptive statistical techniques were used.

To examine the correlation between the variables the Pearson's correlation test was used. For analyzing the normally distributed data the independent samples *t* test, Chi-square and Fisher's exact test, one-way analysis of variance, and LSD were used.

The Q-Q plot for normality was used. With a backward model, logistic regression analysis was performed for related risk factors of GDM. While GDM was taken as the dependent variable, associated risk factors for GDM were considered as independent variables, in this analysis. Using the Mann-Whitney U and Kruskal-Wallis tests, non-parametric data were analyzed.

For quantitative data and percentage of qualitative data, results are shown as arithmetic mean ± standard deviation. P < 0.05 was considered as statistically significant. To compare the means between groups, the paired samples *t* test was used. In logistic regression analysis, odds ratio (OR) (95% CI) was used (5).

## 3. Result

The mean age of the participants was 31.24 ± 4.78 yr (range 19-45 yr). The mean maternal, BMI was 24.81 ± 4.01 kg/m${}^{2}$ (range 16.66-46.33 kg/m${}^{2}$).

The characteristics of the study population are outlined in Table I. After omitting the diabetic patients and incomplete data, 613 participants remained. An impaired GTT was detected in 143 (23.3%) patients. For BS controlling, 72 (11.7%) patients used insulin and/or metformin; the remainder (71) were controlled by diet modification.

Among the 613 women, 461 (75.2%) had a first-trimester FBS ≤ 90 mg/dl and 152 (24.8%) had FBS > 90. Of those with FBS ≤ 90 mg/dl, 83 (18%) patients had impaired GTT and 38 (8.2%) used insulin and/or metformin to control their BS.

Among those with FBS > 90 mg/dl, 60 (39.5%) patients had impaired GTT and 34 (22.4%) used insulin and/or metformin to control their BS. GDM prevalence was higher in the first-trimester FBS > 90 mg/dl group (p < 0.001) (Table II).

A comparison of the GDM and the NGT groups demonstrated significant differences in the maternal age, first-trimester FBS, third-trimester vitamin D levels, maternal platelet count, maternal BMI (before the 12^th^ wk of gestation), weight gain during the pregnancy, and the history of gestational complications in previous pregnancies (Table III).

No association was seen between GDM and NT, anemia, TSH, family history of diabetes mellitus, infant sex, and infant weight.

The overall prevalence of pregnancy complications (namely PTL, PROM, preeclampsia, and IUGR) was not significantly different between diabetic and non-diabetic patients (p = 0.530). However, the history of gestational complications in previous pregnancies (e.g., preeclampsia, hypertension, IUGR, PTL, placenta abruption, and GDM) was associated with a higher chance of GDM in the current pregnancy (p = 0.004). A multivariate analysis using a multiple logistic regression model was perform to determine the independence of these variables.

In this analysis, GDM was significantly and independently associated with older maternal age, higher first-trimester FBS, the history of gestational complications in previous pregnancy, lower third-trimester vitamin D level, and higher maternal platelet count (p < 0.01). (Table IV). These risk factors were independent predictors of GDM.

**Table 1:** Characteristics of the study population


**Variables**		**Patients**
Maternal age (yr)*		31.24 ± 4.78 (19–45)
Infant's gender **		
	**Boy**	57.5%
	**Girl**	42.5%
Infant's birth weight (gr)*		3278.26 ± 417.99 (1200–4480)
**Infant's gestational age at birth (wk)**		38.33 (31–42)
Infant's gestational age at birth (wk) **		
	**< 37 **	6.7%
	**≥ 37 **	93.3%
Delivery type **		
	**Cesarean**	87.4%
	**NVD**	22.6%
Maternal BMI (kg/m ${}^{2}$)*		24.81 ± 4.01 (16.66–46.33)
Weight gain during pregnancy (kg)*		14.69 ± 5.26 (1–37)
Impaired GTT***		143 (23.3%)
*Data presented as Mean ± SD (mi-max), **Data presented as percentages, ***Data presented as n (%), Yr: Year, gr: Gram, wk: Week, NVD: Normal vaginal delivery, BMI: Body mass index, kg/m${}^{2}$: kilogram/metere${}^{2}$, kg: Kilogram, GTT: Glucose tolerance test		

**Table 2:** Prevalence of GDM and drug-controlled GDM in Iranian pregnant women according to their first-trimester FBS levels during their first trimester


	**FBS ≤ 90 **	**FBS > 90 **	**Total**	**P-value**
**Having GDM or Not**				
**Non-GDM**	378 (80.9)/(82)	92 (19.1)/(60.5)	470 (76.7)	
**GDM **	83 (57.9)/(18)	60 (42.1)/(39.5)	143 (23.3)	
**Total**	461 (75.2)/(100)	152 (24.8)/(100)	613 (100)	<brow>-3</erow> < 0.001${}^{*}$
**Drug-controlled GDM**				
**Non-drug use**	423 (78.2)/(91.8)	118 (21.8)/(77.6)	541 (88.3)	
**Drug use **	38 (52.8)/(8.2)	34 (47.2)/(22.4)	72 (11.7)	
**Total**	461 (75.2)/(100)	152 (24.8)/(100)	613 (100)	<brow>-3</erow> < 0.001${}^{*}$
Data presented as n (%). Pearson's correlation test. *Significant difference between two groups, FBS: Fasting blood sugar, GDM: Gestational diabetes mellitus				

**Table 3:** Clinical and metabolic characteristics of study groups


	GDM* (n = 143)	Non GDM* (n = 470)	**P-value**
Maternal age (yr)*	32.62 ± 5.1	30.82 ± 4.61	< 0.001a
Maternal weight (before the 12^th^ wk of gestation) [kg]*	67.59 ± 14.28	65.64 ± 10.43	0.078
Maternal BMI (before the 12^th^ wk of gestation) [kg/m ${}^{2}$]*	25.74 ± 4.73	24.53 ± 3.72	0.006a
FBS (1^st^ trimester) [mg/dl]*	88.92 ± 11.9	83.77 ± 7.28	< 0.001a
TSH (1^st^ trimester) [UI/dl]*	2.4 ± 0.7	2.35 ± 0.79	0.793
Vit D (1^st^ trimester) [ng/dl]*	20.98 ± 16.04	20.99 ± 15.67	0.999
Hemoglobin (1^st^ trimester) [mg/dl]*	13 ± 0.81	12.94 ± 1.24	0.597
Platelet count (1^st^ trimester) [/μl]*	259510.79 ± 52194.22	234164.44 ± 54438.74	< 0.001a
TSH (3^rd^ trimester) [μUI/dl]*	2.56 ± 0.59	2.28 ±0.22	0.097
Vit D (3^rd^ trimester) [ng/dl]*	29.30 ± 12.22	33.75 ± 15.37	0.005a
Hemoglobin (3^rd^ trimester) [mg/dl]*	12.17 ± 1.08	12.14 ± 1.32	0.881
Platelet count (3^rd^ trimester) [/μl]*	244290.91 ± 65190.17	215744.68 ± 56738.81	0.003a
Maternal weight gain during pregnancy [kg]*	12.48 ± 5.77	15.37 ± 4.91	< 0.001
Maternal weight gain (2^nd^ trimester) [kg]*	5.76 ± 2.80	7.06 ± 2.78	< 0.001a
Maternal weight gain (3^rd^ trimester) [kg]*	3.75 ± 1.36	5.41 ± 1.42	< 0.001a
Infant weight [gr]*	3288.19 ± 331.71	3275 ± 442.99	0.748
NT [mm]*	1.76 ± 0.61	1.68 ± 0.52	0.194
Past pregnancy obstetrical history [%]**	75 (52.4%)	182 (38.7%)	0.004a
Infant sex (male)***	53%	58.9%	0.232
Delivery type (cesarean)***	88.3%	87.2%	0.770
Overall prevalence of pregnancy complications**	16 (11.2%)	62 (13.2%)	0.530
Family history of diabetes mellitus**	64 (44.8%)	184 (39.1%)	0.244
*Data presented as Mean ± SD, **Data presented as percentages, ***Data presented as n (%). Pearson correlation test. P-value was considered significant < 0.05, GDM: Gestational diabetes mellitus, BMI: Body mass index, FBS: Fasting blood sugar, TSH: Thyroid stimulating hormone, Vit D: Vitamin D, NT: Nuchal translucency			

**Table 4:** Logistic regression analysis of risk factors for GDM among 613 pregnant women


**variable**	**B**	**SE**	**Beta**	**T**	**P-value**
**Maternal age (yr)**	0.02	0.007	0.23	3.071	0.002*
**Maternal BMI (before the 12^th^ week of gestation) [kg/m ${}^{2}$]**	–0.008	0.007	–0.078	–1.115	0.267
**FBS (1^st^ trimester) [mg/dl]**	0.009	0.003	0.202	2.755	0.007*
**Platelet count (1^st^ trimester) [/μl]**	0.000	–0.036	–0.428	0.669
**Vit D (3^rd^ trimester) [ng/dl]**	–0.006	0.002	–0.223	–3.220	0.002*
**Platelet count (3^rd^ trimester) [/μl]**	0.000	0.221	2.594	0.01*
**Maternal weight gain during pregnancy [kg]**	–0.007	0.01	–0.099	–0.759	0.449
**Maternal weight gain (during 2^nd^ trimester) [kg]**	–0.012	0.015	–0.082	–0.824	0.411
**Maternal weight gain (during 3^rd^ trimester) [kg]**	–0.011	0.015	–0.071	–0.735	0.463
**Past pregnancy obstetrical history [%]**	0.103	0.057	0.124	1.81	0.027*
*P-value < 0.05 was considered as significant. logistic regression analysis, GDM: Gestational diabetes mellitus, BMI: Body mass index, FBS: Fasting blood sugar, Vit D: Vitamin D					

## 4. Discussion

This research represents a population-based study in which factors associated with GDM were investigated. We found that higher FBS in the first trimester, lower vitamin D level in the second trimester, maternal age, maternal BMI (before the 12^th^ wk of gestation), the history of gestational complications in previous pregnancy, and weight gain during the pregnancy were associated with GDM. There were no association between GDM and NT, anemia, status of thyroid function, family history of diabetes mellitus, and neonate weight. We found that the history of gestational complications in the previous pregnancy, including preeclampsia, hypertension, IUGR, PTL, placenta abruption, and GDM, were associated with a higher chance of GDM in the current pregnancy. We also found a strong graded association between fasting glucose level in the first trimester and abnormal GTT; first-trimester higher FPG levels among pregnant women constitutes an independent risk factor for the development of GDM. Maternal older age, higher first-trimester FBS, lower third-trimester vitamin D level, the history of gestational complications in previous pregnancy, and higher maternal platelet count were independent predictors of GDM.

GDM is a growing health challenge in many parts of the world. Certain populations are particularly vulnerable to growing this circumstance due to genetic, social, and environmental factors. Parallel with increased incidence of obesity in adolescent and adult women, gestational diabetes is detected more frequently by obstetricians (14). GDM involves serious, short- and long-term consequences for both the neonate and the mom, including macrosomia, caesarean section, birth trauma, a predisposition to obesity, metabolic syndrome, and diabetes mellitus later in life (15). Early prediction of women at high risk for developing GDM is likely to enhance pregnancy outcomes because it can minimize later development of GDM or its later maternal and perinatal complications by applying effective intervention through diet and exercise adjustment and medical therapies earlier in pregnancy. Unfortunately, for early prediction of GDM, there are no uniform worldwide indices (8).

In our study, 23.3% of patients had impaired GTT known as GDM. Insulin and/or metformin were needed to control diabetes in 11.7% of patients, while in the remaining the disease was controlled solely through diet and exercise modification. Previous surveys have reported GDM prevalence between 1 and 14% with extensive-ranging variations among countries. Moreover, within the identical area, the prevalence of GDM varies on the subject of ethnicity, methods of data collection, selection, the screening methods, and the diagnostic criteria adopted (7, 9). Jafari-Shobeiri and colleagues reported “the prevalence of GDM in Iranian population to be 3.41% (the highest and the lowest prevalence rates were 18.6% and 1.3%, respectively)" (3). A higher incidence in our study is probably because of relatively higher-risk population attending to our tertiary referral clinics. Also, as we use the newest guideline (75g 2hOGTT) one-step strategy (Based on the American Diabetic Association) (12) to predict GDM, the prevalence is predictably higher. In the Shahbazian and colleagues survey, the prevalence of GDM was 29.9% among pregnant women. The authors attributed their findings to the application of newer guidelines (International association of diabetes and pregnancy study groups criteria) (4) which we believe is the case in our study.

In our survey, the first-trimester fasting glucose level was an independent predictor of GDM. “higher first-trimester fasting glucose levels, within the normoglycemic range were an independent risk factor for the development of GDM in young pregnant women." The use of FPG as a GDM screening test offers some advantages over the glucose challenge test because it is easy to administer, nicely tolerated, affordable, reliable, reproducible, and has been reported to differ little during gestation. In order to avoid GDM complications, first-trimester FBS would help detect and treat seemingly healthy women with GDM early in pregnancy (8, 11).

Based on the findings of our study, maternal age is strongly associated with GDM. Therefore, the older age of mother results in the greater chance of GDM (9, 16-18).

In our study, the prevalence of GDM had a positive association with maternal BMI and obesity. Many surveys have reported that maternal BMI and obesity are associated with a higher prevalence of GDM and are independent risk factors for growing GDM. Due to inappropriate lifestyle, the rate of obesity is increasing which in turn follows by rising rates of GDM (19-22).

Weight gain during pregnancy has been associated with GDM in many previous surveys (7, 23-25). In our study, diabetic patients underwent counseling by a nutritionist and adopted a strict dietary and exercise regimen, and hence, we saw a lower weight gain in GDM patients compared to the non-GDM group in our study.

This shows the advantage of lifestyle modification on at least some aspects of GDM as expressed in the previous studies (25, 26). In our study, despite tight glycemic control and lower weight gain in diabetic mothers than in non-diabetic mothers, neonatal birth weight was not significantly different between them.

We found that third-trimester low vitamin D levels were associated with increased risk of GDM. Similar to our study, Burris *et al *showed “second-trimester vitamin D levels were inversely associated with glucose levels after 1-hr 50g glucose challenge test" (27). A number of studies also showed a higher prevalence of vitamin D deficiency in pregnancies that complicated with GDM (28-30).

Further, a significant association was found between higher platelet count and GDM. There are several studies supporting this finding (31, 32). It might be attributed to the thrombosis tendency in GDM. The association between GDM and inflammation might be another contribution (31, 32). In contrast to our study, Erikci and colleagues showed that women with GDM had lower platelet counts and higher mean platelet volume (MPV). It might be due to relative small sample size in their study (34 GDM and 45 normal pregnancies) (33).

We found no association between GDM and TSH. Yang and coworkers reported that a low level of FT4 is an independent risk factor for GDM; however, TSH level and TPO antibody did not predict GDM, in line with our findings (16).

In our study, patients with a history of a previously complicated pregnancy, including preeclampsia, hypertension, abortion, placental abruption, PTL, IUGR, GDM, hypothyroidism, and premature rupture of membranes, had a greater risk of developing GDM. These previous history of pregnancy complications have common risk factors consisting of increased maternal age, nulliparity, multiple gestation pregnancies, and an increased prepregnancy BMI. Vascular endothelial dysfunction is considered to be the underlying pathophysiology of these conditions. Although previous obstetric history has not yet been considered a key risk factor for subsequent development of GDM, it is imperative to clarify the relationship of the two. Lee and colleagues found that the risk of GDM in the second pregnancy was further increased by preeclampsia and GDM in the first pregnancy, which support our findings (10). This finding suggests that patients with a positive past gestational history should be considered as high risk for GDM and should be screened earlier for GDM.

In this survey, because of close observation and control of GDM patients, the prevalence of pregnancy complications, including hypertension, preeclampsia, intra uterus fetal death, and PTL was not significantly different between the GDM and non-GDM groups and were lower than previous studies, which is best attributed to the early diagnosis and management of diabetes in the pregnancy.

It should be mentioned that this study has some constraints. First, this is a retrospective study that naturally depends on the medical records only. Second, the status of participants before the 16^th^ wk of gestation was not considered in our study. Third, this is a single-center study with relatively small sample size. Further studies with larger sample sizes and long-term follow-ups need to be performed to verify our findings and focus on gathering more information about the effectiveness of early intervention for high-risk pregnant women to prevent GDM incidence and its complications.

## 5. Conclusion

The aim of early GDM prediction is to identify women at risk for adverse outcome of pregnancy and improve the prognosis. The lack of consensus concerning diagnostic criteria, however, made it difficult for women with GDM to be identified early. This survey provides information on early GDM prediction to make local and evidence-based decisions.

Patients with a history of previous complicated pregnancy, including preeclampsia, abortion, placental abruption, PTL, GDM, hypothyroidism, IUGR, and premature rupture of membranes, had a greater risk of developing GDM. This finding suggests that patients with a positive past gestational history should be considered high risk for GDM and should be screened earlier for GDM. This survey suggests women with higher fasting first-trimester glucose levels which is in normal range according to the oral glucose tolerance test (OGTT) are at an increased risk of developing GDM during pregnancy. Our study suggests that higher initial FPG which is in normal level and the history of gestational complications in previous pregnancy could be used as an indicator for predicting the development of GDM.

##  Conflict of Interest

The authors have no conflict of interest with respect to the research, authorship, and/or publication of this article.
